# Hematological patterns and histopathological assessment of Miniature Pigs in the experiments on human mesenchymal stem cell transplantation

**DOI:** 10.7150/ijms.53036

**Published:** 2021-01-15

**Authors:** Young-Bum Son, Dinesh Bharti, Saet-Byul Kim, Eun-Yeong Bok, Sang-Yeob Lee, Han-Jang Ho, Sung-Lim Lee, Gyu-Jin Rho

**Affiliations:** Department of Theriogenology and Biotechnology, College of Veterinary Medicine and Research Institute of Life Science, Gyeongsang National University, Jinju, Republic of Korea.

**Keywords:** Human mesenchymal stem cells, miniature pigs, CBC test, blood chemistry, lymphocyte subset.

## Abstract

**Background:** Multipotent and immune privileged properties of mesenchymal stem cells (MSCs) were investigated for the treatment of various clinical diseases. For the years, many researches into the animal studies evaluated human stem cell therapeutic capacity related to the regenerative medicine. However, there were limited reports on immune privileged properties of human MSCs in animal studies. The present study investigated hematological and biochemical parameter and lymphocyte subset in mini-pigs following human MSCs transplantation as a means of validation of reliability that influence the animal test results.

**Methods:** The miniature pigs were transplanted with human MSCs seeded with scaffold. After transplantation, all animals were evaluated by CBC, biochemistry and lymphocyte subset test. After 9 weeks, all pigs were sacrificed and organs were histologically analyzed.

**Results:** CBC test showed that levels of RBC were decreased and reticulocyte, WBC and neutrophil were increased in transient state initially after transplantation, but returned to normal value. The proportion of B lymphocyte and cytotoxic T cell were also initially enhanced within the normal range temporarily. The female and male miniature pigs showed normal ranges for blood chemistry assessments. During the 9 weeks post-operative period, the animals showed a continuous increase in body weight and length. Furthermore, no abnormal findings were observed from the histological analysis of sacrificed pigs.

**Conclusions:** Overall, miniature pigs transplanted with human MSCs seeded with scaffold were found to have physiologically similar results to normal animals. This result might be a reliable indicator of the animal experiments using miniature pigs with human MSCs.

## Introduction

Mesenchymal stem cells (MSCs) had been considered as prominent therapeutic agents in regenerative medicine. It was reported that MSCs had been utilized in various studies due to their peculiar qualities including self-renewability, multi-differentiation potential and immune-privileged 1, 2. To date, various preclinical animal experiments had been performed when inventing human stem cell therapies 1, 3, 4, 5, 6. And many results had been reported for regeneration of various tissues 1, 7, 8, 9. Additionally, valuable outputs concerning application of cellular densities, induced biological responses, adverse effects, sites and methods of cell administration in clinical trials had been gained. However, limited studies had been performed on physiological and immunological status in animals.

Many animal studies had been executed before use in clinical trials to confirm efficiency and safety on MSCs therapy. Various kinds of animals were used for evaluating preclinical investigations, including mouse, rat, rabbit, goat, dog and miniature pig 1, 3, 4, 5, 6. And they reported that human MSCs could be employed to regenerate various kinds of tissues such as neuron, bone, teeth and cartilage. Furthermore, along with the verification of reliability of these results on animal studies, another crucial aspect was the validation of physiological and immunological responses by transplanted human MSCs. Several studies had been reported to prove these aspects, however most studies were performed on mice and rats 10, 11.

Similarly, physiological and genetic characteristics to human, animal studies in miniature pig were significant value in preclinical studies 12, 13, 14. Therefore, further progress demanded studies on immunogenicity of transplanted cells into miniature pigs to assess the stability and reliability of these preclinical studies. Moreover, studies of hematological and histological changes following transplantation of human MSCs into miniature pigs had not been reported. So, the evaluation of physiological and immunological changes *in vivo* study was essential.

Generally, the value of complete blood counts and biochemistry were objective to evaluate physiological states 15, 16. We analyzed changes of hematologic and biochemistry values. Specially, red blood cell (RBC) and reticulocyte were changed by bleeding by invasive surgery and then return to normal status 17, 18. In addition, white blood cells (WBC) and neutrophil had been supposed markers for early response of inflammation and bacterial infection 19, 20. Therefore, we dealt with a comparison of changes in hematologic and biochemical values ​​in normal controls for 9 weeks pre and post human MSCs transplantation. Interestingly, the lymphocyte subset was an indicator for the presence of leukocyte antigens 21, 22. These antigens were normally found on WBCs, either on the surface of white blood cells or inside cells. Several researches had been reported that antigens with abnormal characteristics were present in abnormal immune status 23, 24. After that, we investigated physiological and immunological status of those transplanted human MSCs about body length and weight in miniature pig and also confirmed histological evaluation. The aim of this study was to evaluate the hematological status, lymphocyte subtype variation and histopathological analysis of miniature pigs which were transplanted human MSCs.

## Materials and Methods

### Animals (Miniature pig)

Female and male miniature pigs aged from 12 to 18 months and weighing approximately 25 kg were used in this study. The miniature pigs were kept in healthy condition, without any disease during the experiments. All animal experiments were conducted after getting approval from the Ethics Committee of Gyeongsang National University (GNU-160913-p0047). In this study, a total of 22 miniature pigs (female: 15, male: 7) were used to perform CBC and serum biochemistry analysis. To establish lymphocyte subset, 17 miniature pigs (female: 10, male: 7) were used. In addition, the six miniature pigs (female: 3, male: 3) were used for the evaluation of human MSCs transplantation. All animals were kept for at least 3 weeks before the experiment and were anesthetized with zoletil 50 (Virbac, Carros, France, KR; 10 mg/kg) and xylazine hydrochloride (Bayer Korea, Ansan, KR; 4 mg/kg). After anesthetizing, submandibular and mandibular body was exposed by incision. Further, to evaluate the *in vivo* efficacy of MSCs, a total of 1 x 10^7^ human MSCs with scaffold were transplanted onto the bone defects. The incision site was sutured with two layers (Ethicon, Somerville, NJ, USA). After 9 weeks, animals were sacrificed and organs were sampled for histological analysis.

### Blood collection

Blood samples of miniature pig were collected through internal jugular vein at 1 to 3 weeks intervals. For preventing experimental effects of the animal's physiological changes, 25 ~ 40 ml blood sample was collected based on the recommended one time bleeding volume and recovery times 25. For analysis of complete blood counts (CBC), blood chemistry and lymphocyte subset, samples were collected in the EDTA-containing tubes and used for experiment within 6 hours.

### Hematological analysis

Analysis of CBC was performed for total 15 parameters using an Procyte DX hematology analyzer (IDEXX, westbrook, ME, USA) : red blood cell, hematocrit, hemoglobin, mean corpuscular volume (MCV), mean corpuscular hemoglobin (MCH), mean corpuscular hemoglobin concentration (MCHC), reticulocyte, white blood cell, neutrophil, lymphocyte, monocyte, eosinophil, basophil, platelet and platelet distribution width (PDW).

Serum chemistry analyses were performed using IDEXX Catalyst Dx (IDEXX, westbrook, ME, USA) that included the examination of glucose, bun, creatinine, phosphorus, calcium, NA^+^, K^+^, Cl^-^, osmolality, total protein, albumin, globulin, alanine aminotransferase (ALT), alkaline phosphatase (ALKP), gamma(γ)-glutamyl transferase (GGT), total bilirubin and total cholesterol.

For lymphocyte subset, we isolated peripheral blood mononuclear cells (PBMCs) through density gradient centrifugation using Ficoll paque (Sigma, St. Louis, MO, USA) 22, 24. These assessments were analyzed on a flow cytometry (BD FACSVerseTM, BD biosciences, USA) following previously described methods 22. In brief, PBMCs were directly stained with following multi-monoclonal antibodies anti CD3 PerCP-Cy^TM^5.5, anti CD4 Alexa Flour 647 and anti CD8α FITC. CD3+CD4- NK cells, CD3+CD4+ helper T cells, CD4-CD8α+ cytotoxic T cells and CD3-CD4- B cells were identified according previous reports.

### Measurement of body length and weight

The recording of body length and weight was performed on miniature pigs from pre- to post- human MSCs transplantation. Briefly, miniature pigs were anesthetized and body weight and length from scapula to rump were measured at the following times: pre-transplantation (1 week, 2 weeks and 3 weeks) and post-transplantation (1 day, 1 week, 2 weeks, 3 weeks, 4 weeks, 6 weeks and 9 weeks).

### Histopathologic assessment

Both female and male miniature pigs were sacrificed at 9 weeks after transplantation. For histopathologic assessment, 6 kinds of organ tissues (liver, spleen, thymus, lymph node, left and right kidney cortex with medulla and lung) were fixed with 10% formalin and embedded in paraffin. After that, H&E staining was performed. All tissue sections were observed under a light microscope.

### Statistical analysis

All data were analyzed by One-way analysis of variance (ANOVA) using SPSS 23 (IBM), and Tukey's post hoc test was performed for multiple comparisons. The data were represented as means ± standard error (SE). The differences were considered significant when *p* < 0.05.

## Results

### Complete blood counts and biochemistry analysis

**Figure 1:**
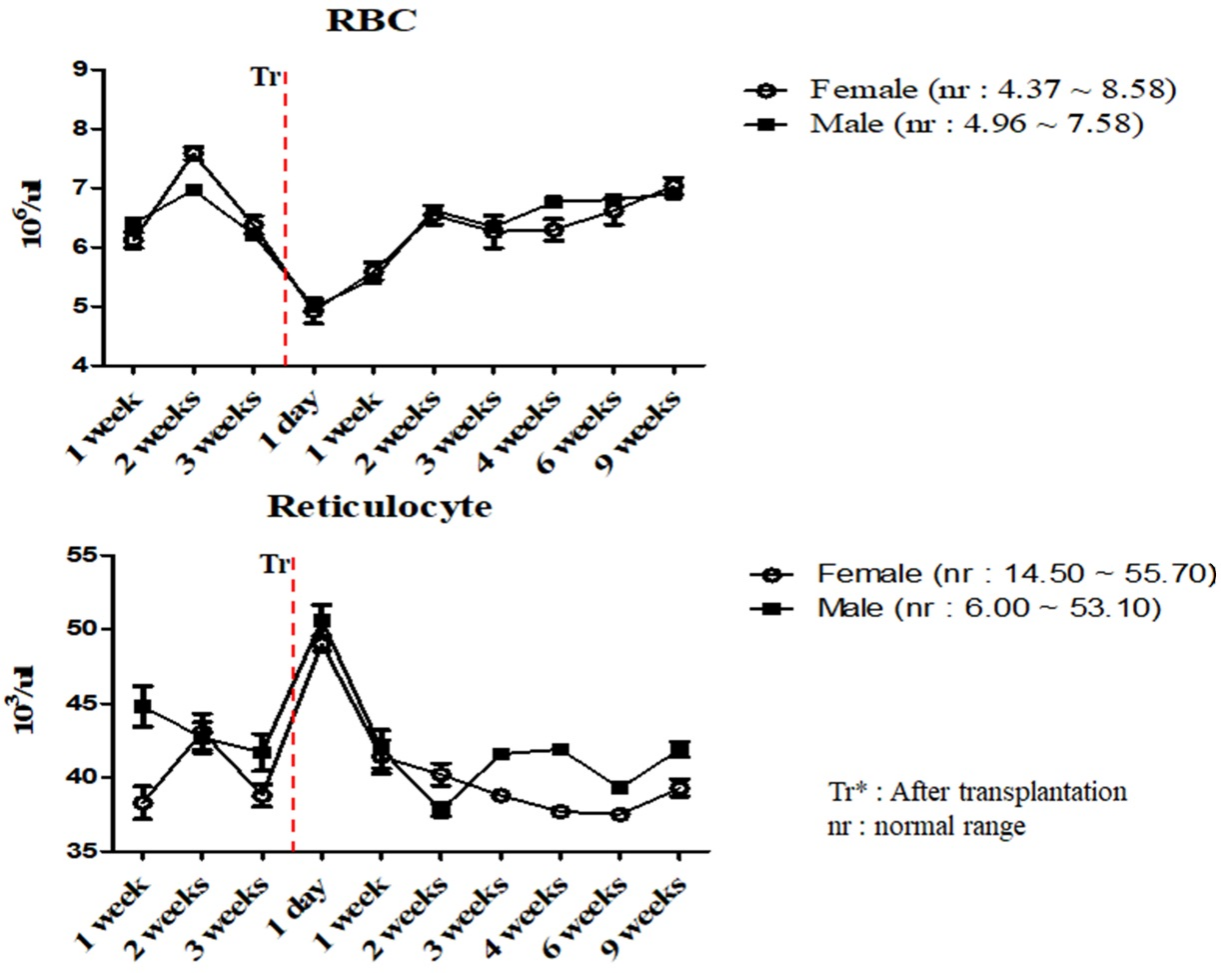
Mean values of RBC and reticulocytes were analyzed on MSCs transplanted miniature pigs. Values are represented by the mean ± SEM of six pigs. RBC, red blood cells. Tr*: After transplantation. nr: normal range.

**Figure 2:**
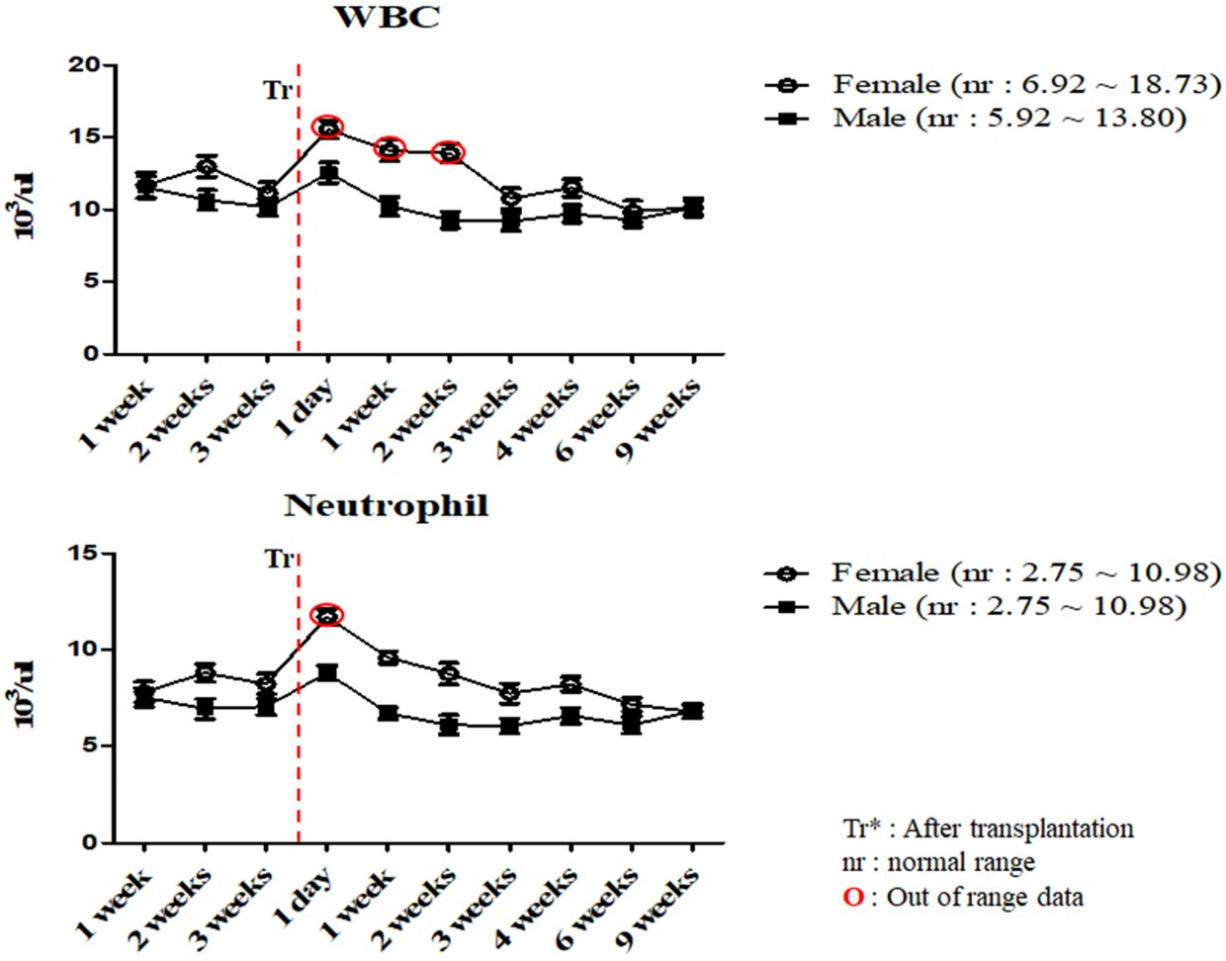
Mean values of WBC and neutrophil were analyzed on MSCs transplanted miniature pigs. Values are represented by the mean ± SEM of six pigs. WBC, white blood cells. Tr: After transplantation. nr: normal range. **O**: Out of range data

To investigate the changes in the physiological and immunological status of miniature pigs following human MSCs transplantation, we performed CBC test of normal control and experimental groups. In this study, we used 12 to 18 months old miniature pigs. A total of 22 miniature pigs (female: 15, male: 7) were used to determine CBC data and serum chemistry test for constructing control group data (Table S1, S2). Most of CBC results were similar to control pigs in MSCs transplanted group except for a few values (Table 1, 2). The concentration of RBC was temporally decreased post-transplantation but gradually increased, and reticulocyte concentration was increased on day 1 post-transplantation, however showed a similar pattern from the 1 week post-transplantation (Figure 1). These changes were observed within normal range. In addition, concentration of WBC was increased for 2 weeks post-transplantation, and neutrophil was increased 1 day post-transplantation, but then returned to normal range in female pigs (Figure 2). The values of blood chemistry were estimated, and all the results were within control group range in MSCs transplanted group (Table 3, 4).

### Lymphocyte subset

**Figure 3:**
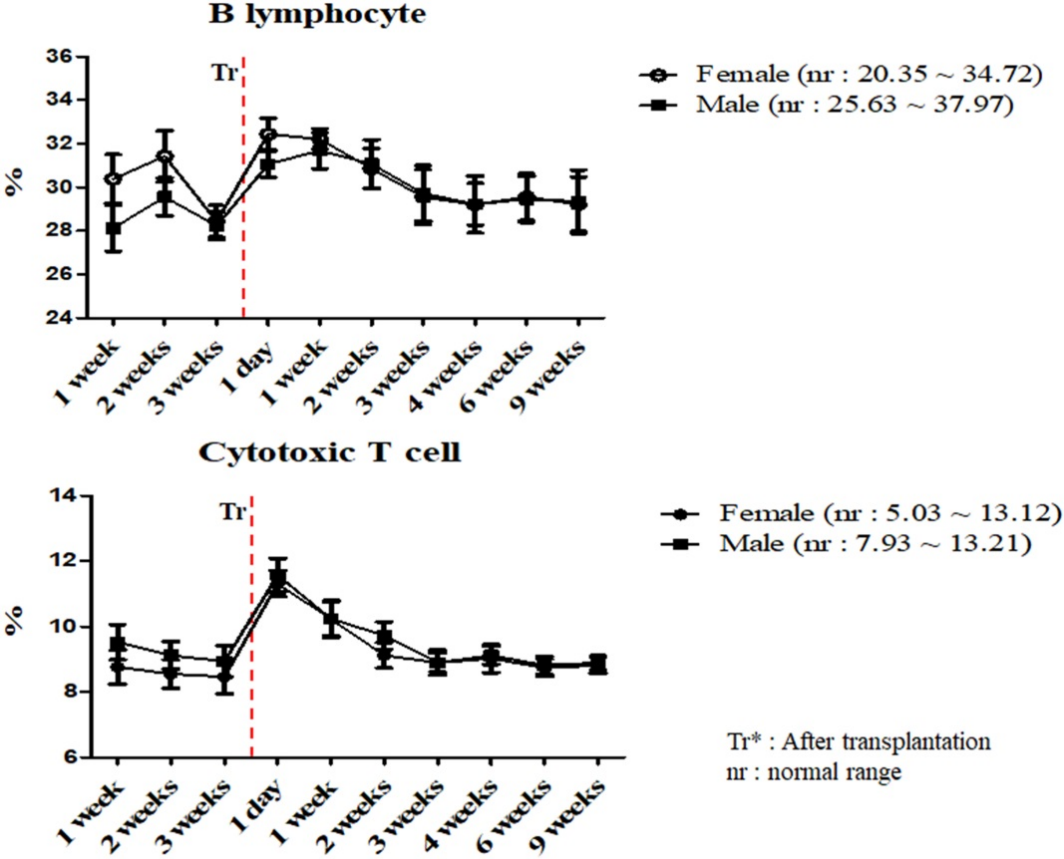
Mean values of B lymphocyte and cytotoxic T cell were analyzed on MSCs transplanted miniature pigs. Values are represented by the mean ± SEM of six pigs. Tr: After transplantation. nr: normal range.

A total of 17 miniature pigs (female: 10, male: 7) were used to establish mean values of lymphocyte subset for establishing control group data (Table S3). The results of helper T lymphocyte, NK cell, B lymphocyte and cytotoxic T cell were within normal limits in MSCs transplanted group (Table 5). Interestingly, the ratio of B lymphocyte and cytotoxic T cell were increased on day 1 post-transplantation, and then decreased in both pigs (Figure 3). The frequency of B lymphocyte and cytotoxic T cell were within normal ranges.

### Values of body weight and length

We measured the mean body weight and length in MSCs transplanted miniature pigs. The mean body weight and length gradually increased during the 3 weeks MSCs pre-transplantation, and also increased over the 9 weeks post-transplantation (Table 6).

### Histopathology examination

**Figure 4:**
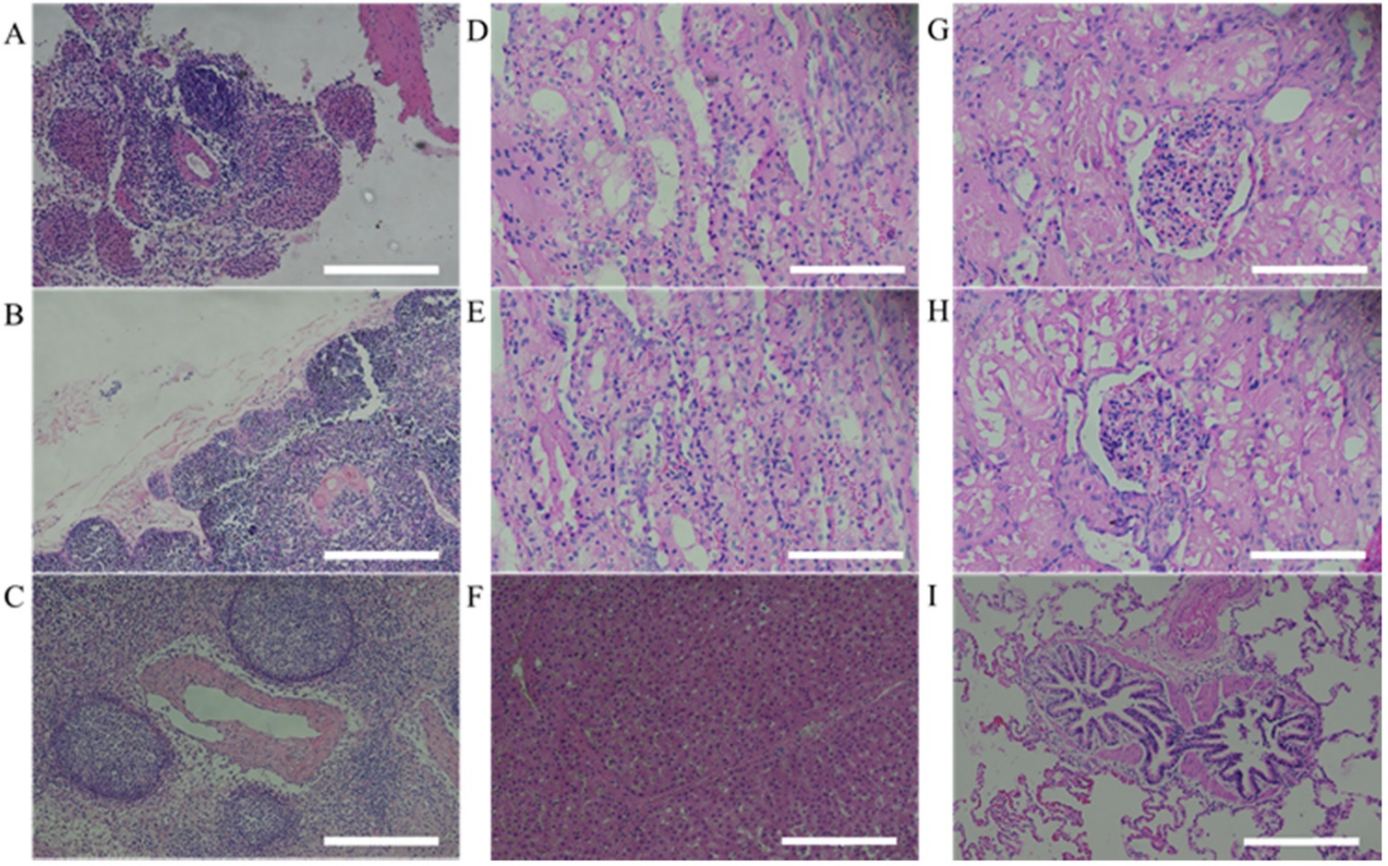
Histopathological assessment of MSCs transplanted miniature pig organs. H&E staining was performed in miniature pigs. All organs revealed normal state. (A: spleen, B: thymus, C: lymph node, D: left kidney cortex, E: right kidney cortex, F: left kidney medulla, G: right kidney medulla, H: lung, I: liver). H&E staining, Haematoxylin and Eosin staining. Scale bar = 250 μm.

After 9 weeks from human MSCs transplantation, miniature pigs were sacrificed and evaluated for histological analysis. According to the results of previous hematological patterns and values of body weight & length, normal physiological and immune status was observed in miniature pigs. Histopathologic examination provided the background for these results. For evaluating immune response in pig, the organs (spleen, thymus and lymph node) were stained with H&E and the structure of organs was identified as normal state (Figure 4). Furthermore, when the human stem cell with scaffold were moved *in vivo*, the organs (Left kidney cortex, right kidney cortex, right kidney cortex, right kidney medulla, lung and liver) were evaluated to confirm histopathological assessments and showed normal state (Figure 4).

## Discussion

Many animal experiments had been carried out to investigate human MSCs based therapy 1, 3, 4, 5, 6. *In vivo* models and studies of preclinical investigation provided strategies for regenerative medicine. However, most studies regarding physiological and immunological status changes caused by human stem cell transplantation were limited to mice. Miniature pigs were suitable animal species because of their similar characteristics to human and therefore several studies had also been reported in this regard 26, 27. But, the matter of fact is that that it could be much appropriate to check the reliability of such results using miniature pigs, which is still lacking. The purpose of this study was to confirm the reliability of the results of animal experiments by hematologic and histological analysis of miniature pig following human MSCs transplantation.

In accordance with the CBC and serum biochemistry results, changes were discovered in some parameters in MSCs transplanted group and control although most of values remained similar compared to control. Bleeding from invasive surgery for MSCs transplantation caused decreasing of the RBC values and increasing of the reticulocyte temporally within normal ranges 17, 18. One of the crucial factors in this study was the changing patterns of the WBC and neutrophil values. Leukocytes are essential part of the immune system and play different roles depending on the type and WBC was also clinically useful to determine indicators of infection 19, 20, 23, 28. Leukocytes could be divided into neutrophil, lymphocyte, monocyte, eosinophil and basophil according to differentiation process, size and shape, especially, neutrophil number is increased after infection or inflammatory reaction 19, 20. As shown in Table 2 and Figure 2, mean WBC and neutrophil values was increased temporarily in female miniature pigs after MSCs transplantation, however the values were observed as normal over time compared to control group. These results could be explained on the fact that despite the temporary changes after procedure, the immunological changes such as inflammation were not observed. Our studies also indicated that the mean values of blood chemistry parameter of MSCs transplanted group was similar to control groups such as BUN, Creatinine and Osmolality. The BUN and Creatinine were parameters that reflect renal perfusion and kidney filtration 29, 30. As shown in Table 4, mean values of BUN and Creatinine showed similar pattern. These results revealed that dehydration due to bleeding and stress did not affect kidney function. Additionally, the bleeding which was a decrease in isotonic fluid and dehydration by stress did not affect the osmolality. Overall, results demonstrated that these miniature pigs might be physiologically normal.

The adaptive immune system is regulated by lymphocytes, especially T and B lymphocytes 31. These cells are stimulated by dendritic cells and monocytes 21. B lymphocytes recognizes specific antigens and releases antibodies into the fluids 32. In addition, T lymphocytes are differentiated into several types. Cytotoxic T lymphocytes cause removal of damaged cells and Helper T lymphocytes supports immune response against specific antigens 33, 34, 35. We evaluated the frequency of cytotoxic and helper T cell and B lymphocyte in MSCs transplanted group. According to our study, these results were similar to control and changed within normal range and further stated the normal immune status of the pigs.

In the previous studies, human MSCs did not cause an immune response even after transplantation into mini pigs 36, 37. However, compared to hematological and *in vitro* assessment demonstrating the immunogenicity of MSCs, studies on histopathological analysis after MSCs transplantation are limited. 38, 39. Therefore, we evaluated lymphoid organs which regulates the immune systems and kidney and lung which is filter organs of transplanted stem cells. The lymphoid organs are divided into primary lymphoid organs and secondary lymphoid organs. The thymus where T cell maturation occurs in primary lymphoid organ and lymph node which have nodules and possess immune response to antigens and spleen that maintains the immune system by generating lymphocytes are secondary lymphoid organs.

The histopathological analysis of these three kinds of organs evaluated normally, so we determined that the mini pigs were in a normal immune condition. These results indicated that there were no immune rejection reactions by the lymphoid organs when human MSCs were. It had been reported that after transplantation, most MSCs are filtered by liver, kidney and lung 40. Keeping these things in mind, in the present study, we performed H&E staining of these organs and they did not show any abnormal findings. Therefore, we determined that transplanted MSCs were safely homed to the mandibular bone defect.

In conclusion, we demonstrated that the mean values of WBC, neutrophil and reticulocytes were increased and RBC were decreased after human MSCs transplantation. However, these changes appeared temporarily and were mainly observed within normal ranges, suggesting that it was due to the non-specific reaction of external stress occurred because of the experimental procedures. Furthermore, hematological patterns and histopathological assessment were normal in human MSCs transplanted group compared to control. Taken together, the human MSCs transplanted into miniature pigs did not cause an immunological rejection and normal physiological conditions were observed. These results supported the stability and reliability of pre-clinical studies in mini pigs using human MSCs.

## Supplementary Material

Supplementary tables.

## Figures and Tables

**Table 1:** Mean values of complete blood counts in miniature pigs depending on MSCs transplantation.

Time	Sex	RBC (10^6^/ul)	Hematocrit (%)	Hemoglobin (g/dl)	MCV (fl)	MCH (pg)	MCHC (g/dl)	Reticulocyte (10^3^/ul)
1 week	Female	6.12 ± 0.13	30.70 ± 1.51	9.50 ± 0.35	60.00 ± 1.20	18.60 ± 0.26	30.90 ± 0.15	38.30 ± 1.11
	Male	6.40 ± 0.21	32.40 ± 1.17	10.00 ± 0.41	60.00 ± 1.17	18.50 ± 0.21	30.90 ± 0.13	44.80 ± 1.37
2 weeks	Female	7.58 ± 0.11	45.00 ± 1.28	13.90 ± 0.38	59.40 ± 1.05	18.30 ± 0.13	30.90 ± 0.21	43.10 ± 1.21
	Male	6.96 ± 0.17	41.30 ± 1.63	12.80 ± 0.37	59.30 ± 0.89	18.40 ± 0.21	31.00 ± 0.17	42.70 ± 1.03
3 weeks	Female	5.87 ± 0.15	36.40 ± 1.96	11.60 ± 0.32	62.00 ± 1.06	19.80 ± 0.27	31.90 ± 0.13	38.80 ± 0.75
	Male	5.21 ± 0.11	30.70 ± 1.82	9.80 ± 0.28	60.50 ± 1.14	18.40 ± 0.18	31.40 ± 0.28	41.70 ± 1.26
1 day (Tr^*^)	Female	4.92± 0.21	30.10 ± 2.02	9.50 ± 0.29	56.60 ± 0.79	18.60 ± 0.26	31.60 ± 0.32	49.00 ± 0.48
	Male	4.99 ± 0.17	29.70 ± 1.63	9.70 ± 0.23	59.50 ± 0.83	18.40 ± 0.25	31.00 ± 0.37	50.60 ± 1.06
1 week (Tr^*^)	Female	5.59 ± 0.15	32.50 ± 1.54	10.20 ± 0.17	58.80 ± 0.74	18.30 ± 0.21	32.40 ± 0.26	41.40 ± 1.13
	Male	5.46 ± 0.32	32.60 ± 1.53	12.20 ± 0.09	57.70 ± 1.16	18.40 ± 0.16	31.90 ± 0.17	41.90 ± 1.27
2 weeks (Tr^*^)	Female	6.54 ± 0.16	36.50 ± 1.47	12.00 ± 0.29	58.10 ± 1.26	18.20 ± 0.17	31.40 ± 0.34	40.20 ± 0.76
	Male	6.62 ± 0.31	38.20 ± 1.06	12.30 ± 0.33	59.70 ± 1.23	18.90 ± 0.13	31.60 ± 0.12	37.80 ± 0.43
3 weeks (Tr^*^)	Female	6.26 ± 0.27	35.60 ± 1.52	11.30 ± 0.31	56.90 ± 0.91	18.10 ± 0.21	31.70 ± 0.18	38.80 ± 0.21
	Male	6.34 ± 0.25	38.70 ± 1.39	11.90 ± 0.36	60.60 ± 1.03	18.40 ± 0.18	31.40 ± 0.31	41.60 ± 0.18
4 weeks (Tr^*^)	Female	6.29 ± 0.18	35.70 ± 1.28	11.40 ± 0.28	56.80 ± 0.84	18.10 ± 0.13	31.90 ± 0.25	37.70 ± 0.15
	Male	6.76 ± 0.21	39.30 ± 1.21	12.40 ± 0.25	58.10 ± 0.73	18.30 ± 0.17	31.60 ± 0.11	41.90 ± 0.32
6 weeks (Tr^*^)	Female	6.61 ± 0.23	36.30 ± 2.08	11.80 ± 0.23	54.90 ± 0.76	17.90 ± 0.12	32.50 ± 0.27	37.50 ± 0.24
	Male	6.80 ± 0.15	35.10 ± 2.16	11.00 ± 0.15	58.50 ± 0.71	18.50 ± 0.21	31.30 ± 0.23	39.30 ± 0.39
9 weeks (Tr^*^)	Female	7.03 ± 0.14	37.00 ± 1.37	11.90 ± 0.19	63.30 ± 1.03	17.70 ± 0.15	31.00 ± 0.19	39.30 ± 0.57
	Male	6.90 ± 0.16	36.50 ± 1.52	12.10 ± 0.24	57.90 ± 0.68	18.40 ± 0.24	31.60 ± 0.24	41.90 ± 0.49
Control range	Female	4.37 ~ 8.58	25.20 ~ 58.80	8.30 ~ 18.10	56.60 ~ 68.50	18.40 ~ 22.50	30.40 ~ 34.10	14.50 ~ 55.70
	Male	4.96 ~ 7.58	30.70 ~ 45.00	9.50 ~ 13.90	57.70 ~ 66.50	18.30 ~ 22.00	30.90 ~ 33.00	6.00 ~ 53.10

Values are represented by the mean ± SEM. Each group contains 3 miniature pigs (female: 3, male: 3). Tr*: After transplantation

**Table 2:** Mean values of complete blood counts in miniature pigs depending on MSCs transplantation.

Time	Sex	WBC (10^3^/ul)	Neutrophil (10^3^/ul)	Lymphocyte (10^3^/ul)	Monocyte (10^3^/ul)	Eosinophil (10^3^/ul)	Basophil (10^3^/ul)	Platelet (10^3^/ul)	PDW (%)
1 week	Female	11.68 ± 0.87	7.81 ± 0.53	3.54 ± 0.12	0.2 ± 0.02	0.12 ± 0.01	0.01 ± 0.01	305.00 ± 10.12	18.90 ± 0.26
	Male	11.52 ± 0.75	7.53 ± 0.49	3.43 ± 0.11	0.43 ± 0.03	0.11 ± 0.01	0.02 ± 0.01	326.00 ± 9.87	19.10 ± 0.23
2 weeks	Female	12.97 ± 0.73	8.80 ± 0.45	3.34 ± 0.14	0.55 ± 0.01	0.19 ± 0.01	0.01 ± 0.01	249.00 ± 10.52	21.30 ± 0.21
	Male	10.66 ± 0.68	6.94 ± 0.52	2.81 ± 0.08	0.56 ± 0.02	0.33 ± 0.02	0.01 ± 0.01	214.00 ± 10.36	20.80 ± 0.18
3 weeks	Female	11.15 ± 0.71	8.23 ± 0.51	2.42 ± 0.09	0.47 ± 0.01	0.02 ± 0.01	0.01 ± 0.01	200.00 ± 11.21	21.00 ± 0.25
	Male	10.16 ± 0.59	7.05 ± 0.43	2.69 ± 0.11	0.40 ± 0.03	0.01 ± 0.01	0.01 ± 0.01	198.00 ± 10.36	20.50 ± 0.17
1 day (Tr^*^)	Female	15.52 ± 0.62Δ	11.69 ± 0.41Δ	3.23 ± 0.10	0.44 ± 0.02	0.10 ± 0.01	0.06 ± 0.01	203.00 ± 9.89	21.30 ± 0.15
	Male	12.53 ± 0.72	8.80 ± 0.37	3.13 ± 0.07	0.40 ± 0.04	0.01 ± 0.01	0.01 ± 0.01	268.00 ± 11.21	21.10 ± 0.21
1 week (Tr^*^)	Female	14.08 ± 0.72Δ	9.59 ± 0.32	3.63 ± 0.05	0.48 ± 0.01	0.21 ± 0.01	0.10 ± 0.01	204.00 ± 10.36	20.60 ± 0.23
	Male	10.22 ± 0.64	6.71 ± 0.31	3.11 ± 0.06	0.36 ± 0.01	0.02 ± 0.01	0.02 ± 0.01	206.00 ± 10.25	20.80 ± 0.12
2 weeks (Tr^*^)	Female	13.88 ± 0.63Δ	8.76 ± 0.56	4.36 ± 0.04	0.56 ± 0.04	0.16 ± 0.02	0.02 ± 0.01	272.00 ± 9.84	20.20 ±0.15
	Male	9.26 ± 0.57	6.11 ± 0.49	2.74 ± 0.12	0.40 ± 0.02	0.14 ± 0.01	0.03 ± 0.01	198.00 ± 9.56	21.00 ±0.16
3 weeks (Tr^*^)	Female	10.75 ± 0.71	7.74 ± 0.52	2.66 ± 0.06	0.29 ± 0.01	0.03 ± 0.01	0.03 ± 0.01	278.00 ± 9.42	21.20 ±0.18
	Male	9.17 ± 0.64	6.05 ± 0.37	2.41 ± 0.05	0.17 ± 0.01	0.27 ± 0.02	0.03 ± 0.01	206.00 ± 10.21	21.10 ±0.23
4 weeks (Tr^*^)	Female	11.50 ± 0.61	8.22 ± 0.39	3.05 ± 0.07	0.20 ± 0.01	0.02 ± 0.01	0.01 ± 0.01	306.00 ± 9.81	20.50 ± 0.22
	Male	9.70 ± 0.59	6.58 ± 0.41	2.71 ± 0.09	0.49 ± 0.03	0.19 ± 0.02	0.03 ± 0.01	234.00 ± 10.26	20.70 ± 0.13
6 weeks (Tr^*^)	Female	9.86 ± 0.73	7.15 ± 0.36	2.43 ± 0.10	0.18 ± 0.02	0.09 ± 0.01	0.01 ± 0.01	236.00 ± 9.72	21.00 ± 0.15
	Male	9.28 ± 0.51	6.11 ± 0.45	2.75 ± 0.07	0.39 ± 0.03	0.01 ± 0.01	0.01 ± 0.01	186.00 ± 9.62	21.00 ± 0.21
9 weeks (Tr^*^)	Female	10.14 ± 0.48	6.79 ± 0.32	2.98 ± 0.06	0.29 ± 0.02	0.07 ± 0.01	0.01 ± 0.01	236.00 ± 9.63	21.30 ± 0.20
	Male	10.13 ± 0.62	6.83 ± 0.37	2.75 ± 0.04	0.53 ± 0.02	0.01 ± 0.01	0.01 ± 0.01	159.00 ± 9.58	21.00 ±0.14
Control range	Female	6.92 ~ 18.73	2.75 ~ 10.98	2.24 ~ 4.68	0.17 ~ 0.78	0.01 ~ 0.36	0.01 ~ 0.03	120.00 ~ 327.00	17.40 ~ 22.40
	Male	5.92 ~ 13.80	2.75 ~ 10.98	1.76 ~ 3.49	0.07 ~ 0.56	0.01 ~ 0.33	0.01 ~ 0.10	198.00 ~ 326.00	17.40 ~ 21.30

Values are represented by the mean ± SEM. Each group contains 3 miniature pigs (female: 3, male: 3). Tr*: After transplantation. **Δ** : Out of range data.

**Table 3:** Mean values of serum chemistry test in miniature pigs depending on MSCs transplantation.

Time	Sex	Glucose (mg/dl)	BUN (mg/dl)	Creatinine (mg/dl)	Phosphorus (mg/dl)	Calcium (mg/dl)	Na+ (mmol/l)	K+ (mmol/l)	Cl- (mmol/l)	Osmorality (mOsm/kg)
1 week	Female	94.00 ± 3.78	23.00 ± 1.01	0.97 ± 0.01	6.20 ± 0.05	9.50 ± 0.05	138.00 ± 0.75	3.50 ± 0.12	99.00 ± 0.41	277.00 ± 1.21
	Male	93.00 ±3.21	23.00 ±0.97	0.98 ± 0.03	6.20 ± 0.03	9.60 ± 0.07	138.00 ± 0.73	3.50 ± 0.25	99.00 ± 0.37	277.00 ± 1.05
2 weeks	Female	93.00 ±2.79	21.00 ± 0.85	0.96 ± 0.02	6.00 ± 0.02	9.70 ± 0.03	145.00 ± 0.75	4.00 ± 0.26	99.00 ± 0.38	282.00 ± 1.23
	Male	87.00 ±3.05	21.00 ± 1.13	0.98 ± 0.03	5.90 ± 0.04	9.30 ± 0.05	140.00 ± 0.62	4.60 ± 0.13	96.00 ± 0.27	282.00 ± 1.15
3 weeks	Female	74.00 ±3.12	22.00 ±0.83	0.98 ± 0.05	6.30 ± 0.06	9.40 ± 0.04	144.00 ± 0.71	3.70 ± 0.15	98.00 ± 0.15	281.00 ± 0.85
	Male	66.00 ±3.30	13.00 ±0.92	0.96 ± 0.03	6.30 ± 0.05	9.10 ± 0.04	138.00 ± 0.81	3.50 ± 0.17	95.00 ± 0.38	278.00 ± 0.73
1 day (Tr*)	Female	76.00 ±2.86	22.00 ±1.05	0.94 ± 0.07	6.10 ± 0.04	9.30 ± 0.03	145.00 ± 0.56	3.50 ± 0.09	98.00 ± 0.41	285.00 ± 0.72
	Male	80.00 ±2.57	20.00 ±0.76	0.96 ± 0.02	5.50 ± 0.03	9.10 ± 0.06	139.00 ± 0.73	4.00 ± 0.08	96.00 ± 0.38	281.00 ± 1.02
1 week (Tr*)	Female	97.00 ±2.68	22.00 ±0.84	0.95 ± 0.03	6.20 ± 0.05	9.00 ± 0.05	138.00 ± 0.68	5.20 ± 0.11	95.00 ± 0.31	281.00 ± 0.89
	Male	89.00 ±2.76	18.00 ±1.03	0.97 ± 0.02	5.60 ± 0.04	9.40 ± 0.04	142.00 ± 0.53	3.60 ± 0.15	96.00 ± 0.36	282.00 ± 0.93
2 weeks (Tr*)	Female	67.00 ±3.01	22.00 ±0.92	0.95 ± 0.03	6.10 ± 0.02	9.10 ± 0.03	143.00 ± 0.46	4.80 ± 0.16	99.00 ± 0.35	286.00 ± 0.86
	Male	87.00 ±3.13	20.00 ±0.68	0.98 ± 0.05	6.30 ± 0.02	9.20 ± 0.05	139.00 ± 0.68	5.10 ± 0.07	96.00 ± 0.28	283.00 ± 0.72
3 weeks (Tr*)	Female	98.00 ±2.74	20.00 ± 0.75	0.95 ± 0.03	5.80 ± 0.03	9.00 ± 0.02	141.00 ± 0.72	3.30 ± 0.08	97.00 ± 0.27	280.00 ± 0.68
	Male	72.00 ±2.76	15.00 ± 1.01	0.98 ± 0.05	5.40 ± 0.04	8.40 ± 0.02	143.00 ± 0.42	4.60 ± 0.05	103.00 ± 0.29	2823.00 ± 0.59
4 weeks (Tr*)	Female	88.00 ±2.59	21.00 ± 0.91	0.96 ± 0.02	5.20 ± 0.04	9.40 ± 0.03	145.00 ± 0.37	3.50 ± 0.03	96.00 ± 0.32	288.00 ± 0.42
	Male	76.00 ±3.01	12.00 ± 0.86	0.96 ± 0.02	6.50 ± 0.03	8.70 ± 0.03	140.00 ± 0.41	4.60 ± 0.07	97.00 ± 0.31	278.00 ± 0.57
6 weeks (Tr*)	Female	89.00 ±3.12	19.00 ± 1.04	0.99 ± 0.02	5.70 ± 0.02	8.60 ± 0.02	142.00 ± 0.52	4.20 ± 0.11	97.00 ± 0.18	281.00 ± 0.46
	Male	71.00 ±2.86	15.00 ± 0.91	0.97 ± 0.02	5.40 ± 0.01	9.10 ± 0.04	144.00 ± 0.63	3.50 ± 0.21	95.00 ± 0.28	283.00 ± 0.59
9 weeks (Tr*)	Female	84.00 ±3.16	21.00 ± 0.95	0.98 ± 0.03	6.10 ± 0.02	9.30 ± 0.04	143.00 ± 0.48	4.30 ± 0.17	96.00 ± 0.24	280.00 ± 0.42
	Male	79.00 ±2.72	17.00 ± 0.83	0.96 ± 0.01	5.60 ± 0.01	9.00 ± 0.03	144.00 ± 0.42	5.30 ± 0.09	99.00 ± 0.26	289.00 ± 0.37
Control range	Female	64.00 ~ 159.00	7.00 ~ 23.00	0.80 ~ 1.00	4.70 ~ 6.40	8.60 ~ 9.70	138.00 ~ 145.00	3.10 ~ 5.90	91.0 ~ 99.00	270.00 ~ 286.00
	Male	71.00 ~ 94.00	12.00 ~ 28.00	0.90 ~ 1.00	5.20 ~ 7.30	8.50 ~ 9.70	138.00 ~ 146.00	3.30 ~ 5.40	95.00 ~ 103.00	277.00 ~ 293.00

Values are represented by the mean ± SEM. Each group contains 3 miniature pigs (female: 3, male: 3). Tr*: After transplantation

**Table 4:** Mean values of serum chemistry test in miniature pigs depending on MSCs transplantation.

Time	Sex	Total protein (g/dl)	Albumin (g/dl)	Globulin (g/dl)	ALT (U/L)	ALKP (U/L)	GGT (U/L)	Total bilirubin (mg/dl)	Total cholesterol (mg/dl)
1 week	Female	7.20 ± 0.10	3.70 ± 0.04	3.50 ± 0.03	68.00 ± 0.84	59.00 ± 1.36	37.00 ± 1.25	0.40 ± 0.01	31.00 ± 1.52
	Male	7.20 ± 0.11	3.70 ± 0.03	3.50 ± 0.04	67.00 ± 1.26	95.00 ± 1.08	41.00 ± 1.13	0.40 ± 0.01	42.00 ± 1.05
2 weeks	Female	7.70 ±0.15	3.80 ± 0.04	3.90 ± 0.06	64.00 ± 1.06	63.00 ± 1.16	35.00 ± 1.06	0.10 ± 0.01	44.00 ± 1.38
	Male	6.70 ±0.13	3.00 ± 0.07	3.70 ± 0.04	89.00 ± 0.92	93.00 ± 1.12	38.00 ± 1.39	0.20 ± 0.01	68.00 ± 1.47
3 weeks	Female	7.90 ±0.14	3.70 ± 0.02	4.00 ± 0.05	58.00 ± 0.79	61.00 ± 1.39	54.00 ± 0.62	0.20 ± 0.01	35.00 ± 1.29
	Male	7.20 ±0.08	3.10 ± 0.05	4.10 ± 0.05	84.00 ± 1.03	91.00 ± 1.82	38.00 ± 0.89	0.10 ± 0.01	80.00 ± 1.37
1 day (Tr^*^)	Female	7.90 ±0.13	3.50 ± 0.03	4.10 ± 0.06	66.00 ± 1.05	68.00 ± 1.91	39.00 ± 1.36	0.10 ± 0.01	35.00 ± 1.33
	Male	7.00 ±0.14	3.00 ± 0.06	4.00 ± 0.08	87.00 ± 0.46	95.00 ± 0.83	46.00 ± 1.05	0.10 ± 0.01	79.00 ± 1.25
1 week (Tr^*^)	Female	7.40 ±0.07	3.40 ± 0.07	4.00 ± 0.04	69.00 ± 0.75	97.00 ± 1.49	58.00 ± 1.32	0.10 ± 0.01	34.00 ± 1.16
	Male	7.30 ±0.09	3.20 ± 0.03	4.10 ± 0.03	61.00 ± 0.89	149.00 ± 2.13	68.00 ± 0.89	0.10 ± 0.01	57.00 ± 1.34
2 weeks (Tr^*^)	Female	7.80 ±0.11	3.90 ± 0.03	4.00 ± 0.03	69.00 ± 1.27	94.00 ± 2.69	51.00 ± 1.63	0.60 ± 0.02	36.00 ± 1.21
	Male	7.70 ±0.10	3.40 ± 0.02	4.30 ± 0.04	70.00 ± 1.31	148.00 ± 3.16	68.00 ± 1.08	0.10 ± 0.01	62.00 ± 1.06
3 weeks (Tr^*^)	Female	6.80 ±0.13	3.30 ± 0.04	3.50 ± 0.03	59.00 ± 1.29	93.00 ± 4.12	61.00 ± 1.37	0.10 ± 0.01	32.00 ± 1.32
	Male	7.90 ±0.07	3.80 ± 0.03	4.10 ± 0.07	75.00 ± 1.28	129.00 ± 2.46	57.00 ± 0.98	0.40 ± 0.02	64.00 ± 1.07
4 weeks (Tr^*^)	Female	7.00 ±0.05	3.70 ± 0.03	3.30 ± 0.05	56.00 ± 1.32	106.00 ± 3.14	56.00 ± 1.34	0.10 ± 0.01	52.00 ± 1.12
	Male	7.20 ±0.11	3.50 ± 0.06	3.70 ± 0.07	79.00 ± 1.46	119.00 ± 3.59	49.00 ± 1.28	0.30 ± 0.01	91.00 ± 1.10
6 weeks (Tr^*^)	Female	7.00 ±0.05	3.60 ± 0.05	3.30 ± 0.06	69.00 ± 1.05	102.00 ± 4.26	43.00 ± 1.09	0.10 ± 0.01	39.00 ± 1.05
	Male	7.00 ±0.04	3.40 ± 0.05	3.60 ± 0.08	72.00 ± 1.62	149.00 ± 5.21	62.00 ± 1.67	0.10 ± 0.01	58.00 ± 1.08
9 weeks (Tr^*^)	Female	6.90 ±0.07	3.30 ± 0.08	3.00 ± 0.11	63.00 ± 1.52	119.00 ± 4.92	51.00 ± 1.68	0.10 ± 0.01	36.00 ± 1.06
	Male	7.20 ±0.06	3.20 ± 0.06	4.00 ± 0.10	81.00 ± 1.73	139.00 ± 5.11	48.00 ± 1.53	0.40 ± 0.02	98.00 ± 1.03
Control range	Female	6.70 ~ 8.00	2.90 ~ 4.40	3.00 ~ 4.10	26.00 ~ 89.00	22.00 ~ 180.00	15.00 ~ 63.00	0.10 ~ 9.00	32.00 ~ 89.00
	Male	6.40 ~ 7.90	3.00 ~ 3.90	3.00 ~ 4.50	54.00 ~ 89.00	39.00 ~ 153.00	35.00 ~ 75.00	0.10 ~ 0.40	35.00 ~ 105.00

Values are represented by the mean ± SEM. Each group contains 3 miniature pigs (female: 3, male: 3). Tr*: After transplantation

**Table 5:** Mean values of lymphocyte subset in miniature pigs depending on MSCs transplantation.

Time	Sex	Helper T lymphocyte (%)	NK cell (%)	B lymphocyte (%)	Cytotoxic T cell (%)
1 week	Female	23.24 ± 1.07	4.58 ± 0.24	30.37 ± 1.12	8.76 ± 0.52
	Male	29.12 ± 1.13	4.23 ± 0.21	28.11 ± 1.05	9.52 ± 0.54
2 weeks	Female	23.54 ± 1.28	4.61 ± 0.18	31.42 ± 1.17	8.55 ± 0.43
	Male	29.06 ± 0.82	4.06 ± 0.16	29.56 ± 0.86	9.12 ± 0.42
3 weeks	Female	20.53 ± 0.76	5.79 ± 0.15	28.43 ± 0.73	8.46 ± 0.52
	Male	23.20 ± 0.59	5.18 ± 0.05	28.23 ± 0.64	8.94 ± 0.48
1 day (Tr^*^)	Female	24.70 ± 1.02	3.59 ± 0.13	32.44 ± 0.72	11.31 ± 0.39
	Male	31.10 ± 1.01	4.01 ± 0.17	31.06 ± 0.59	11.57 ± 0.51
1 week (Tr^*^)	Female	23.85 ± 0.85	2.47 ± 0.15	32.21 ± 0.47	10.23 ± 0.57
	Male	34.80 ± 1.01	4.08 ± 0.21	31.68 ± 0.83	10.24 ± 0.53
2 weeks (Tr^*^)	Female	30.08 ± 0.92	4.87 ± 0.19	30.85 ± 0.92	9.12 ± 0.39
	Male	33.45 ± 0.84	4.12 ± 0.23	31.07 ± 1.11	9.72 ± 0.42
3 weeks (Tr^*^)	Female	23.74 ± 0.72	3.92 ± 0.16	29.54 ± 1.26	8.90 ± 0.38
	Male	38.72 ± 1.06	2.72 ± 0.14	29.72± 1.28	8.90 ± 0.29
4 weeks (Tr^*^)	Female	25.16 ± 0.62	2.38 ± 0.25	29.20 ± 1.31	9.02 ± 0.43
	Male	31.26 ± 0.39	3.09 ± 0.18	29.22 ± 0.95	9.12 ± 0.28
6 weeks (Tr^*^)	Female	28.93 ± 1.05	2.31 ± 0.11	29.54 ± 1.08	8.74 ± 0.27
	Male	31.43 ± 0.75	3.35 ± 0.16	29.46 ± 1.06	8.82 ± 0.27
9 weeks (Tr^*^)	Female	24.44 ± 0.74	3.09 ± 0.09	29.22 ± 1.25	8.81 ± 0.24
	Male	30.26 ± 0.73	3.23 ± 0.15	29.34 ± 1.46	8.89 ± 0.21
Control range	Female	20.53 ~ 38.82	2.23 ~ 6.62	20.35 ~ 34.72	5.03 ~ 13.12
	Male	23.12 ~ 44.12	2.69 ~ 5.63	25.63 ~ 37.97	7.93 ~ 13.21

Values are represented by the mean ± SEM. Each group contains 3 miniature pigs (female: 3, male: 3). Tr*: After transplantation

**Table 6:** Mean values of body weight and length in miniature pigs depending on MSCs transplantation.

Time	Sex	Body weight (kg)	Body length (cm)
1 week	Female	23.23 ± 1.07	82.83 ± 0.31
	Male	21.17 ± 0.61	78.80 ± 0.30
2 weeks	Female	23.50 ± 1.15	83.07 ± 0.45
	Male	21.53 ± 1.15	79.03 ± 0.35
3 weeks	Female	23.87 ± 1.43	84.30 ± 0.26
	Male	21.97 ± 0.71	79.43 ± 0.25
1 day (Tr^*^)	Female	24.30 ± 1.51	85.83 ± 0.32
	Male	22.47 ± 0.65	81.47 ± 0.15
1 week (Tr^*^)	Female	25.03 ± 1.81	87.00 ± 0.30
	Male	22.93 ± 0.55	82.10 ± 0.30
2 weeks (Tr^*^)	Female	25.40 ± 1.87	87.57 ± 0.55
	Male	23.57 ± 0.59	82.37 ± 0.31
3 weeks (Tr^*^)	Female	26.07 ± 1.96	88.00 ± 0.46
	Male	24.03 ± 0.72	82.90 ± 0.40
4 weeks (Tr^*^)	Female	26.50 ± 1.93	88.30 ± 0.50
	Male	24.50 ± 0.95	83.10 ± 0.50
6 weeks (Tr^*^)	Female	27.67 ± 1.47	89.03 ± 0.40
	Male	25.37 ± 1.29	83.37 ± 0.45
9 weeks (Tr^*^)	Female	28.67 ± 0.60	89.70 ± 0.10
	Male	26.13 ± 1.80	83.60 ± 0.50

Values are represented by the mean ± SEM. Each group contains 3 miniature pigs (female: 3, male: 3). Tr*: After transplantation

## Abbreviations

MSC: Mesenchymal stem cell
CBC: Complete blood count
RBC: Red blood cell count
WBC: White blood cell count
EDTA: Ethylenediaminetetraacetic acid
MCV: Mean corpuscular volume
MCH: Mean corpuscular hemoglobin
MCHC: mean corpuscular hemoglobin concentration
PDW: Platelet distribution width
ALT: Alanine aminotransferase
ALKP: alkaline phosphatase
GGT: Gamma(γ)-glutamyl transferase
PBMC: peripheral blood mononuclear cell
CD: Cluster of differentiation
NK cell: Natural killer cell
H&E staining: Haemotoxylin and Eosin staining
BUN: Blood urea nitrogen
