# Supplementary material for: Hematological patterns and histopathological assessment of Miniature Pigs in the experiments on human mesenchymal stem cell transplantation
Source: Int J Med Sci. 2021 Jan 15;18(5):1259–68. doi: 10.7150/ijms.53036 (PMC7847617; doi:10.7150/ijms.53036)
Supplement: Supplementary file 1 — Supplementary tables. [file ijmsv18p1259s1.pdf]

Supplementary Table S1. Mean values of complete blood counts for normal miniature-pigs.

Parameters (unit)	Control female		Control male	
	Mean value	Range	Mean value	Range
RBC (10^6/ul)	5.62 ± 0.32	4.37 ~ 8.58	5.70 ± 0.33	4.96 ~ 7.58
Hematocrit (%)	34.39 ± 2.25	25.20 ~ 58.80	34.44 ± 1.89	30.70 ~ 45.00
Hemoglobin (g/dl)	11.06 ± 0.67	8.30 ~ 18.10	10.96 ± 0.55	9.50 ~ 13.90
MCV (fl)	62.25 ± 1.02	56.60 ~ 68.50	60.54 ± 1.11	57.70 ~ 66.50
MCH (pg)	20.01 ± 0.33	18.40 ~ 22.50	19.30 ± 0.49	18.30 ~ 22.00
MCHC (g/dl)	32.15 ± 0.29	30.40 ~ 34.10	31.84 ± 0.32	30.90 ~ 33.00
Reticulocyte (10^3/ul)	27.66 ± 3.88	14.50 ~ 55.70	25.30 ± 5.84	6.00 ~ 53.10
WBC (10^3/ul)	10.90 ± 1.03	6.92 ~ 18.73	9.99 ± 1.02	5.92 ~ 13.80
Neutrophils (10^3/ul)	5.71 ± 0.74	2.75 ~ 10.98	6.76 ± 1.12	2.75 ~ 10.98
Lymphocyte (10^3/ul)	3.45 ± 0.20	2.24 ~ 4.68	2.78 ± 0.24	1.76 ~ 3.49
Monocyte (10^3/ul)	0.48 ± 0.40	0.17 ~ 0.78	0.34 ± 0.06	0.07 ~ 0.56
Eosinophil (10^3/ul)	0.10 ± 0.03	0.01 ~ 0.36	0.08 ± 0.04	0.01 ~ 0.33
Basophil (10^3/ul)	0.01 ± 0.002	0.01 ~ 0.03	0.04 ± 0.01	0.01 ~ 0.10
Platelet (10^3/ul)	222.6 ± 20.43	120.00 ~ 327.00	263.17 ± 21.19	198.00 ~ 326.00
PDW (%)	20.10 ± 0.34	17.40 ~ 22.40	20.13 ± 0.56	17.40 ~ 21.30

Values are represented by the mean ± SEM. A total of 22 miniature pigs were evaluated (female: 15, male: 7).

Supplementary Table S2. Mean values of serum chemistry test of normal miniature pigs

**Parameters (unit)**	**Control female**		**Control male**	
	**Mean value**	**Range**	**Mean value**	**Range**
Glucose (mg/dl)	93.20 ± 6.65	64 ~ 159	80.00 ± 3.58	71.00 ~ 94.00
Bun (mg/dl)	17.00 ± 1.65	7.00 ~ 23.00	20.86 ± 2.23	12.00 ~ 28.00
Creatinine (mg/dl)	0.77 ± 0.05	0.50 ~ 1.00	0.90 ± 0.07	0.60 ~ 1.10
Phosphorus (mg/dl)	5.68 ± 0.14	4.70 ~ 6.40	6.16 ± 0.23	5.20 ~ 7.30
Calcium (mg/dl)	9.11 ± 0.11	8.60 ~ 9.70	9.16 ± 0.18	8.50 ~ 9.70
Na^+ (mmol/l)	141.33 ± 0.77	138.00 ~ 145.00	143.43 ± 1.07	138.00 ~ 146.00
K^+ (mmol/l)	4.07 ± 0.21	3.10 ~ 5.90	4.00 ± 0.28	3.30 ~ 5.40
Cl^- (mmol/l)	96.20 ± 0.79	91.00 ~ 99.00	98.14 ± 0.94	95.00 ~ 103.00
Osmorality (mOsm/kg)	281.73 ± 1.58	270.00 ~ 286.00	286.00 ± 2.09	277.00 ~ 293.00
Total protein (g/dl)	7.19 ± 0.10	6.70 ~ 8.00	7.34 ± 0.18	6.40 ~ 7.90
Albumin (g/dl)	3.53 ± 0.11	2.90 ~ 4.40	3.60 ± 0.11	3.00 ~ 3.90
Globulin (g/dl)	3.66 ± 0.10	3.00 ~ 4.10	3.77 ± 0.56	3.00 ~ 4.50
ALT (U/L)	53.27 ± 4.99	26.00 ~ 89.00	67.89 ± 3.84	54.00 ~ 89.00
ALKP (U/L)	81.00 ± 13.08	22.00 ~ 180.00	90.00 ± 15.08	39.00 ~ 153.00
GGT (U/L)	41.13 ± 4.43	15.00 ~ 63.00	51.29 ± 5.45	35.00 ~ 75.00
Total bilirubin (mg/dl)	0.29 ± 0.10	0.10 ~ 1.50	0.17 ± 0.04	0.10 ~ 0.40
Total cholesterol (mg/dl)	56.29 ± 4.56	32.00 ~ 89.00	70.50 ± 11.15	35.00 ~ 105.00

Values are represented by the mean ± SEM. A total of 22 miniature pigs were evaluated (female: 15, male: 7).

Supplementary Table S3. Mean values of lymphocyte subset of normal miniature pigs

Parameters (unit)	Control female		Control male	
	Mean value	Range	Mean value	Range
Helper T lymphocyte (%)	29.14 ± 2.13	20.53 ~ 38.82	32.95 ± 2.50	23.12 ~ 44.12
NK cell (%)	4.60 ± 0.39	2.23 ~ 6.62	4.14 ± 0.40	2.69 ~ 5.63
B lymphocyte (%)	30.72 ± 1.38	20.35 ~ 34.72	32.90 ± 1.33	25.63 ~ 37.97
Cytotoxic T cell (%)	8.69 ± 0.73	5.03 ~ 13.12	9.89 ± 0.57	7.93 ~ 13.21

Values are represented by the mean ± SEM. A total of 17 miniature pigs were evaluated (female: 10, male: 7).
